# Current practice of antibiotic utilization for renal colic in the emergency room

**DOI:** 10.1590/S1677-5538.IBJU.2016.0123

**Published:** 2017

**Authors:** Bryan Hinck, Benjamin Larson, Shubha De, Manoj Monga

**Affiliations:** 1 Glickman Urologic and Kidney Institute, Cleveland Clinic Foundation, Cleveland, Ohio, USA;; 2 Alberta Urology Institute, Edmonton, Alberta, Canada

**Keywords:** Antibiotic Prophylaxis, Renal Colic, Emergencies

## Abstract

**Introduction:**

Urinalysis (UA) in the emergency setting for patients with nephrolithiasis produces potentially confusing results leading to treatment of presumed urinary tract infections (UTIs). Our objective was to evaluate the use of antibiotics in patients with nephrolithiasis in a large network of emergency departments (EDs).

**Methods:**

A retrospective analysis of all ED visits associated with an ICD-9 diagnosis of nephrolithiasis and a CT scan between 2010 and 2013 was performed. Urinalysis data, the use of IV and PO antibiotics during the ED visit and at discharge were assessed. The presence of fever, elevated serum WBCs, >5 WBCs per hpf, and/or dip positive nitrites were used as appropriate criteria for antibiotic use.

**Results:**

Urinalysis data were available for 3,518 (70%) of 5,035 patients with an ED diagnosis of nephrolithiasis and CT imaging. Of these visits, 237 patients had positive nitrites (6.7%) and 864 had >5 WBCs per hpf (24.6%) with 158 (4.5%) having both findings for a total of 943 patients. Intravenous antibiotics were given to 244 patients (25.9%) and oral antibiotics were given to 629 patients (66.7 %) with positive UA findings. Of the 2,440 patients with a negative UA and no leukocytosis or fever, 86 patients (3.5%) received IV antibiotics and 533 patients (21.8%) received PO antibiotics upon discharge.

**Conclusions:**

Proper treatment of nephrolithiasis in the ED includes the screening and diagnosis of concomitant UTIs. However, correct interpretation of UA studies is vital to the correct implementation of antibiotic therapy. This study suggests that 1/3 of patients were undertreated and 21.8% were over-treated.

## INTRODUCTION

Though nephrolithiasis continues to be one of the most common disorders treated by urologists, the urologist is rarely the first to diagnose and begin treatment of these patients (1). Due to the acute onset of symptoms, most patients with nephrolithiasis present to the emergency department (ED). A recent report showed there are an average of 700.000 ED visits annually for nephrolithiasis (2). This fact places ED physicians at the frontlines of diagnosis and treatment of nephrolithiasis.

The diagnosis of nephrolithiasis can be complicated by the concurrent presence of urinary tract infections (UTIs) or other urinary anomalies. Urinalysis (UA) is routinely performed as a screening test for nephrolithiasis and UTIs; however, interpretation of these results when both are present can be difficult. The presence of a non-obstructing stone in the ureter or kidney may lead to hematuria as well as inflammation. If present, this will cause positive hemoglobin and leukocyte esterase on UA tests. These findings are often misinterpreted as positive for UTI or for “infected stones” in the ED. The actual incidence of infected stones, from urease-producing bacteria is 1-5% of all kidney stones (3). There does not appear to be standardized criteria for UTI diagnosis and treatment in the ED setting that takes the presence of nephrolithiasis into account.

The issue related to misdiagnosis of UTI in the patients with nephrolithiasis has two faces. First, if patients with actual UTIs in the setting of nephrolithiasis are not properly diagnosed and started on treatment or identified as needing urgent decompression, this may lead to a delay in definitive treatment for these patients. Second, the overtreatment of patients with nephrolithiasis without an actual UTI contributes to the growing number of multidrug resistant pathogens.

Our objective was to evaluate the current use of antibiotics in patients with diagnosis of nephrolithiasis in a large health system network of emergency departments (EDs).

## MATERIALS AND METHODS

A retrospective analysis of all ED visits between December 2010 and March of 2013 at a large health system was conducted under IRB approval. Sixteen emergency department sites including academic programs with residents and community hospitals in two different states were included. Each site utilized the same electronic medical record (EMR), Epic Care (Epic Systems Corp., Madison, Wisconsin) and information was electronically extracted. Visits associated with an ICD-9 diagnosis of nephrolithiasis (592.0, 592.1, and 592.9) with an abdominal and pelvic computed axial tomography (CT) scan performed were evaluated. Inclusion criteria included all adult patients (>18 years old).

Patient demographic data were evaluated. Visual analog pain scores at admission as documented by nursing staff were assessed. Serum laboratory values and urinalysis data were reviewed when available. The administration of intravenous (IV) and oral (PO) antibiotics during ED admission was assessed via entries into the EMR. Antibiotic therapy initiated at discharge was assessed by reviewing medication orders at discharge within the EMR.

Appropriate criteria for antibiotic use was defined as patients found to be febrile (>101 degrees Fahrenheit) and/or UA findings of positive nitrites and/or presence of greater than five white blood cells per high power field (hpf) on microscopy. Given the lack of clear data in the literature regarding UA interpretation in the setting of nephrolithiasis, the definition of pyuria used in this study was based on expert opinion. Whether a urine culture was ordered during the ED visit was evaluated. Patients who had a negative UA or did not have a UA completed with concomitant finding of an elevated serum white blood count (WBC) greater than 11.000/μL or elevated temperature were excluded from the analysis. These patients were considered a “soft indication” for antibiotic administration, where clinical acumen beyond the scope of an EMR data analysis may play a role in decision making.

Statistics were performed using SAS software which included student t-test, ANOVA, and multi-variant analysis. Findings were considered significant if the p value was <0.05.

## RESULTS

Data from 5.035 adult patient visits with ICD-9 codes for nephrolithiasis (592.0, 592.1, and 592.9) and CT imaging performed were identified. Urinalysis data were available for 3.518 patients, representing 70% of patients during the study time period. The mean age of patients with a UA performed was slightly younger at 45 years compared to those whom did not have a UA performed at 46.5. More females had a UA performed. About 5% of patients in both groups were found to have elevated serum WBC >11k/μL. Only a small number (<1%) of patients in both groups were found to have elevated temperatures. Initial pain scores were identical (See Table-1).

**Table 1:** Patient characteristics at ED visits for nephrolithiasis.

	UA Performed (n=3,518)	UA Not Performed (n=1,517)	P value
Age in years (mean, SD)	45.1 (±16.3)	46.5 (±15.6)	0.0034
Male (%)	1738 (49.1%)	845 (55.7%)	<0.0001
Elevated serum WBC > 11,000/μL (%)	191 (5.4%)	63 (4.2%)	0.058
Elevated temperature > 101ºF (%)	11 (0.3%)	4 (0.2%)	0.767
Initial pain score (median, IQR)	8 (7-10)	8 (7-10)	0.504
Gross hematuria on UA (%)	102 (2.9%)	NA	
>3 RBC/hpf on UA (%)	2089 (59.4%)	NA	
Nitrates on UA (%)	237 (6.7%)	NA	
>5 WBC/hpf on UA (%)	864 (24.6%)	NA	
Positive UA (%)*	943 (26.8%)	NA	

* Positive UA = nitrates and/or >5 WBC/hpf

Of the visits with a UA performed (n=3.518), 102 patients (2.9%) had gross hematuria while 2.089 (59.4%) had >3 RBC/hpf on UA microscopy. Among patients with UA performed, 237 patients had positive nitrites (6.7%) and 864 had >5 WBCs per hpf (24.6%). Only 158 patients (4.5%) had both findings. A total of 943 patients were deemed to warrant antibiotic treatment based on UA analysis. 67 of these patients also had concurrent elevated serum WBC >11k/μL or elevated temperature. Of these 943, intravenous antibiotics were given to 244 patients (25.9%) and oral antibiotics were given to 629 (66.7%) at the time of discharge (Table-2).

**Table 2:** Utilization of antibiotics based on UA findings.

	UA Positive (n=943)	UA Negative (n=2,440)*	UA Not Performed (n=1,453)**	P value (ANOVA)
IV antibiotics in ED (%)	244 (25.9%)	86 (3.5%)	51 (3.5%)	<0.0001
Oral antibiotics at discharge from ED (%)	629 (66.7%)	533 (21.8%)	237 (16.3%)	<0.0001

* 135 patients were excluded with elevated temperature or elevated serum WBC as possible indication of antibiotics

** 64 patients were excluded with elevated temperature or elevated serum WBC as possible indication of antibiotics

Of the 2.575 patients with a negative UA finding, 135 patients were noted to have an elevated serum WBC >11k/μL or elevated temperature and therefore treatment may have been warranted, due to either a UTI or another infectious source (i.e. pneumonia), and as such were excluded. Of the remaining 2.443 patients, 86 patients (3.5%) received IV antibiotics and 533 patients (21.8%) received oral antibiotics that were not clearly warranted based on UA results upon discharge from the ED (Table-2).

Among the 1.517 patients seen for nephrolithiasis who did not have a UA performed, 64 were noted to have an elevated serum WBC>11k/μL or elevated temperature and were excluded as treatment may have been warranted due to another source of infection. Of the remaining 1.453 patients, 3.5% of these patients received IV antibiotics and 16.3% received oral antibiotics upon discharge (Table-2).

Looking at the utilization of a confirmatory urine culture, only 570 of the 943 patients with positive UA findings (60.4%) had a culture sent. Of patients that received antibiotics during the ED encounter, 68.5% of those treated with IV antibiotics had a urine culture sent and 51.1% of those treated with PO antibiotics had a urine culture sent.

Multivariate analysis was performed for positive predictors of receiving oral antibiotics at discharge. Male gender, elevated serum WBC, positive UA findings, and having a UA performed were significant predictors for receiving oral antibiotics at discharge (Table-3).

**Table 3:** Multivariate analysis of positive predictors for receiving antibiotics at discharge from ED visit for nephrolithiasis.

Predictive factor	X^2^	P value
UA performed	31.33	<0.0001
>5 WBC on UA	320.29	<0.0001
Positive Nitrites on UA	37.76	<0.0001
VAS Pain score at admission	9.4	0.67
Serum WBC level	24.92	0.0001
Initial temperature	0.58	0.45
Gender	20.49	0.0001
Age	0.50	0.48

## DISCUSSION

The rate of nephrolithiasis is increasing throughout the United States (4-7). With the majority of episodes of kidney stones initially presenting to EDs, the burden of properly diagnosing and managing these patients falls to the ED physicians. One aspect of the diagnosis and management of stone patients is that of screening the urine for possible concomitant UTIs. As this study suggests, the interpretation of UA data may lead to both under and over utilization of antibiotics in these patients. To our knowledge, this is the first study to assess the antibiotic treatment pattern in the ED for patient with nephrolithiasis.

Recognizing the confounding results of a UA in the setting of nephrolithiasis is vital in the initial management of these patients. The presence of leukocyte esterase on dip UA without the presence of nitrites or visible white blood cells on microscopy is not specific for a UTI when inflammation from the stone is likely responsible. There is no consensus in the literature and a paucity of data regarding UA findings in the setting of nephrolithiasis which correlate with UTI. The definition of UA findings suggestive of pyuria used in this study was based on expert opinion, with the aim of accepting a liberal indication for antibiotic use. This definition provides a reference point only, the use of similar criteria in other settings has been shown to be nonspecific (8). Confirmatory urine culture should always be sent from patients with suspicious findings on UA. We found this was properly ordered in less than 2/3 patients.

The data presented in this study suggest that one third of patients presenting to the ED with nephrolithiasis had suggestive findings for pyuria and were not treated with home going antibiotic therapy. The ramifications for correct interpretation of UA results could affect the overall financial burden of nephrolithiasis on healthcare systems (9).

This study also suggests that a significant number of patients were treated with antibiotics that may not have warranted treatment based on UA results. The development of multidrug resistant pathogens has become a challenge to not only the urologist but almost all medical specialties. It is important to educate ED physicians on the interpretation of UA results in patients with nephrolithiasis, on the indications for appropriate antibiotic use and the importance of confirmatory urine cultures.

The limitations of this study include the inherent limitations associated with a large retrospective chart review. The data available were analyzed; however, data such as fever, anti-pyretic use, or antibiotic use prior to arrival was not available. Data regarding specific symptoms such as nausea and vomiting were also not available. Patients were excluded if another possible source of infection was present as evident by elevated WBC (WBC >11k/μL), however for those who were normopenic with a normal UA the rational of the ED provider’s use of antibiotics was not evaluated. Further research is needed to define the precise UA criteria in the setting of nephrolithiasis which correlate with culture proven UTI.

Urologists should work with their local EDs to develop standardized criteria for diagnosing and initial treatment of nephrolithiasis. Patients who are properly diagnosed and have appropriate treatment underway when they are referred to the urologist are more likely to receive prompt and definitive treatment of the kidney stones regardless of which modality of treatment is chosen. Cooperation between the urologist and ED physician can also reduce duplicated investigations and delays, which could improve patient satisfaction and reduce costs. Care pathways may help facilitate the adaptation of best practices.

We propose the following algorithm for the administration of antibiotics in the ED. Firstly, antibiotics should be administered only after a urine sample for culture has been obtained. Secondly, clinical criteria for antibiotic administration include: WBC >15k/μL, UA >10WBC/hpf, temperature >101 degrees F, or nitrites positive on urine dip. Lastly, a urology consultation should be obtained in any of these situations if an obstructing stone is present, as the patient may require emergent decompression of the urinary system.

## CONCLUSION

Proper treatment of nephrolithiasis in the ED includes the screening and diagnosis of potentially dangerous concomitant UTIs. However, correct interpretation of the urinalysis studies in these patients is vital to the correct implementation of antibiotic therapy. If a UTI is suspected based on UA results, a confirmatory culture should be sent and antibiotic treatment should be started. Care should be taken to practice antibiotic stewardship and ensure antibiotics are given only to patients who warrant them to prevent development of multidrug resistant pathogens. This study suggests that one third of patients were under-treated (satisfied criteria for antibiotics did not receive them) and nearly one fourth of patients were over-treated (received antibiotics despite normal urinalysis).
